# Micromachined Tactile Sensor Array for RTSA

**DOI:** 10.3390/mi12111430

**Published:** 2021-11-21

**Authors:** Elliott C. Leinauer, H. Mike Kim, Jae W. Kwon

**Affiliations:** 1 Department of Electrical Engineering and Computer Science, University of Missouri, Columbia, MO 65201, USA; eclmwb@mail.missouri.edu; 2 Department of Orthopedic Surgery, University of Missouri, Columbia, MO 65201, USA; kimhm@health.missouri.edu

**Keywords:** capacitive sensor, glenohumeral joint force, reverse total shoulder arthroplasty (RTSA), polydimethylsiloxane (PDMS), pressure sensor

## Abstract

This work presents a polymer-based tactile capacitive sensor capable of measuring joint reaction forces of reverse total shoulder arthroplasty (RTSA). The capacitive sensor contains a polydimethylsiloxane (PDMS) dielectric layer with an array of electrodes. The sensor was designed in such a way that four components of glenohumeral contact forces can be quantified to help ensure proper soft tissue tensioning during the procedure. Fabricated using soft lithography, the sensor has a loading time of approximately 400 ms when a 14.13 kPa load is applied and has a sensitivity of 1.24 × 10^−3^ pF/kPa at a load of 1649 kPa. A replica RTSA prothesis was 3D printed, and the sensor was mounted inside the humeral cap. Four static right shoulder positions were tested, and the results provided an intuitive graphical description of the pressure distribution across four quadrants of the glenohumeral joint contact surface. It may help clinicians choose a right implant size and offset that best fit a patient’s anatomy and reduce postoperative biomechanical complications such as dislocation and stress fracture of the scapula.

## 1. Introduction

Reverse Total Shoulder Arthroplasty (RTSA) is a nonanatomic constrained prosthesis tailored for conditions which are deemed inoperable by traditional Total Shoulder Arthroplasty (TSA) procedures. With the primary indication for RTSA being rotator cuff tear arthropathy, indications for RTSA have expanded beyond conditions affiliated with rotator cuff defects. These include tumor resection [1], revision shoulder arthroplasty from failed TSA procedures [2,3], irreparable rotator cuff tears leading to pseudoparalysis [4], rheumatoid arthritis [5,6], fracture sequelae [7,8], and complex proximal humeral fractures [9].

Originally devised in the 1970s, RTSA was designed to alleviate common issues that occur in TSA prosthesis, which were predominately loosening of the glenoid component and rotator cuff tear [10]. Proponents of RTSA argued that by placing the socket in the proximal humerus and the prosthetic ball on the glenoid, one could improve active motion and stability without an increased risk of glenoid component loosening [11]. This nonanatomic concept brought upon a spur of mechanical innovations looking to increase the reliability of RTSA procedures. On the forefront of these innovations where three reversed glenohumeral articulations: Mark I, II, and III. These designs focused on modifying the size of the glenoid component and altering the center of rotation (COR). However, these articulations were abandoned due to a significant amount of torque being placed at the scapular fixation site, leading to component loosening and failure [12]. These failures led to a truly groundbreaking design which introduced a fixed and medialized COR, reducing torque on the glenoid component and improving abduction as well as restoring tension of the deltoid fibers by lowering the humerus relative to the acromion [13]. However, this design was far from perfect. Scapular notching was a common consequence of a medialized humerus relative to the scapula. This occurrence can be minimized by laterally offsetting the glenosphere with respect to the glenoid face. Ultimately, this lateralization has been associated with glenosphere failure due to torque transmitted from the upper limb directly to the glenoid baseplate [14]. While these features have minimized poor postoperative outcomes, there are components of this procedure that have pained surgeons for decades. Most notably, ensuring proper soft tissue tensioning during intraoperative procedures, an aspect vital for positive postoperative outcomes, has been challenging.

In addition to adequate humeral and scapular bone stock, a properly functioning deltoid muscle is a prerequisite for a successful RTSA. The deltoid is the primary workhorse for motion in a cuff-deficient shoulder with RTSA, which is attributed to a medialized COR and deltoid lengthening [15]. Without proper deltoid tensioning, there will be inadequate compressive forces across the humeral cup–glenosphere interface. As a result, postoperative complications will arise due to instability since a properly tensioned deltoid compensates for a deficient rotator cuff and provides the stable fulcrum for active elevation and prosthetic stability [16]. If the deltoid is under-tensioned, complications such as dislocation will occur. If the deltoid is over-tensioned, stress fracture of the acromion may occur [13]. Considering each patient has a unique anatomical makeup, adequate deltoid tensioning will be different for each patient. This leaves surgeons with very little guidance to ensure proper deltoid tensioning. In fact, intraoperative determination of deltoid tension may be difficult, guided mostly by subjective surgical experience [8,13]. A tight reduction is the only rubric; with the arm at the side and elbow extended, the conjoined tendon should feel tensioned after reduction [8]. Surgeons have the means to adjust deltoid tensioning as they see fit using various implant designs, placements, and orientations. They do not have a reliable solution to quantify deltoid tension beyond the lateral thrust and bed shuffle test [16], until recently.

Defining the contact loads placed on joints in vivo has interested researchers over the past few decades. Most achieved this by integrating a biocompatible pressure sensor into the fulcrum of the prosthetic limb to monitor the dynamic changes in joint loads over time, such as the inductively powered strain gauges developed to measure the complete contact loads in a total hip joint prosthesis [17]. Using this information could enable surgeons to measure loads in real time, allowing for individualized activity restraint protocols to prolong the longevity of the implant, or the biocompatible capacitive sensor array to be developed to evaluate the three-dimensional strain in a total knee arthroplasty prosthesis [18]. Using this device would allow researchers to fully characterize device loading and prosthesis articulation throughout activities of daily life. Though targeted for different applications, these studies do share one common denominator: they are never implemented in vivo to record the contact forces and moments of joint prosthesis. Eventually, G. Bergmann et al. designed and implemented an inductively powered, six strain-gage sensor arrays into the glenohumeral joint of six right-handed patients to measure contact forces and moments during abduction and forward flexion in 2011 [19]. Their study realized some important observations regarding shoulder joint loads: an additional weight of 2 kg when lifting the arm increases the force by 51–75%; the direction of high forces relative to the humeral head is very constant; and moments in the joint vary more than the forces and their high magnitudes indicate that the implant head is often loaded eccentrically [19]. Notably, this study was conducted using the traditional TSA implant, not RTSA. This changed in 2020 when Farmer et al. implanted a specially designed glenosphere containing four strain gauge force sensors to measure intraoperative glenohumeral contact forces during RTSA [20]. The data gathered from 21 patients established proof of concept in that interoperative joint force measurements can be performed during RTSA procedures [20]. However, the sensor was only designed for intraoperative measurements and had to be removed before the procedure was completed. To this day, no studies that we are aware of have shown the use of a pressure sensor to perform glenohumeral pressure measurements intraoperatively and postoperatively.

The paper introduces a micromachined tactile array capacitive sensor that has the potential to measure soft tissue tensioning of an RTSA prothesis. Previous works such as G. Bergmann et al. and Farmer et al. employed tactile array piezoresistive sensors to characterize joint loads. These sensors have inherent disadvantages for prosthesis applications such as the RTSA. When compared with resistive strain sensors, capacitive sensors offer good linearity with low hysteresis, and are less susceptible to creep and overshoot [21]. Furthermore, strain gauge performance is significantly dependent on ambient temperatures [22]. In addition, strain gauges have poor static loading stability when compared with capacitive sensors [23]. For these reasons, capacitive sensors appear to be better suited for long-term in vivo joint load monitoring due to their excellent sensitivity, static loading stability, and low temperature drift.

Intraoperatively, the sensor has the potential to provide data in real time to surgeons performing the arthroplasty procedure and help ensure adequate soft tissue tensioning and joint loads. Postoperatively, the sensors could give clinicians near-instant feedback when assessing patients with biomechanical complications, allowing for immediate feedback that can warn of prosthesis dislocation or over-tensioning and provide surgeons with reliable point-of-care guidance. This will ultimately prevent complications and improve the clinical outcomes of this increasingly popular procedure.

## 2. Materials and Methods

**Figure 1:**
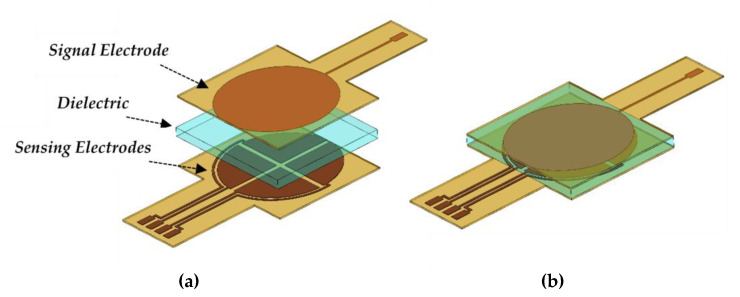
(**a**) Schematic of the capacitive sensor. (**b**) Capacitive sensor after assembly.

The flexible tactile capacitive sensor is composed of four sensing electrodes on the bottom layer, a PDMS dielectric layer, and one signal electrode on the upper layer. Figure 1a,b presents the schematic of the sensor and the sensor’s final layout, respectively. The signal electrode is responsible for supplying the sensor with a sinusoidal signal. Variations of this signal are measured by the four sensing electrodes when interfaced with a data acquisition circuit, allowing the configuration to measure four components of glenohumeral contact forces.

As external compressive forces are applied, the PDMS dielectric layer undergoes elastic deformation. This alters the distance between the upper and lower electrodes to varying degrees and translates to a deviation in capacitance between each of the four sensing electrodes. The total capacitance of each parallel plate electrode pair is defined by the equation of a parallel plate capacitor:

(1) $$C_{x}=\frac{\varepsilon_{0}\varepsilon_{r}A}{d}$$

where *ε_0_* is the vacuum permittivity (8.854 × 10-12 Fm), *ε_r_* is the dielectric constant of PDMS (2.7), *A* is the surface area of the overlapping electrodes, and *d* is the spacing between each parallel plate. The signal electrode has an area of approximately 406.5 mm^2^ and each of the sensing electrodes has an area of approximately 99.1 mm^2^. These parameters alongside the dielectric of PDMS and vacuum permittivity remain constant. Thus, one can conclude by Equation (1) that the capacitance is inversely proportional to the spacing between each overlapping electrode pair. The change in capacitance for each electrode pair can be defined as the mechanical deformation of the dielectric layer while undergoing compression, such that:

(2) $$\Delta C=C_{f}-C_{0}=\varepsilon_{0}\varepsilon_{r}A\left(\frac{d_{0}-d_{f}}{d_{0}d_{f}}\right)$$

*C_f_* is the final capacitance value of the sensor, *C_0_* is the initial capacitance value of the sensor, and ΔC is the change in capacitance under compression. Furthermore, *d_0_* is the distance between overlapping plates before compression (µm) and d_f_ is the distance after compression. Using Equation (2), one can define the sensitivity of the sensor as the change in capacitance over the change in applied pressure:

(3) $$S=\frac{\delta \left(\Delta C\right)}{\delta P}$$

where δP is the intensity of pressure applied across the electrodes in kilopascals (kPa), and *S* is the sensitivity of the sensor (pF/kPa). One can deduce, from Equations (2) and (3), that the sensitivity is dependent on the surface area of the overlapping electrodes, the permittivity of the dielectric, and the degree of deformation of the dielectric layer when compressed. 

While the surface area of the electrodes and permittivity of the dielectric remains constant, the elastic modulus of PDMS can be adjusted. Which, beyond biocompatibility and rapid fabrication, was the reason PDMS was chosen. The elasticity of PDMS is dependent upon the mixing ratio of the pre-polymer PDMS and curing agent. By adjusting this mixing ratio the measurement range and sensitivity of the sensor can be tuned to fit a wide range of pressures [23,24]. In short, increasing the pre-polymer to curing agent ratio results in greater deformation of the dielectric medium with the same applied compression resulting in a greater change in capacitance.

**Figure 2:**
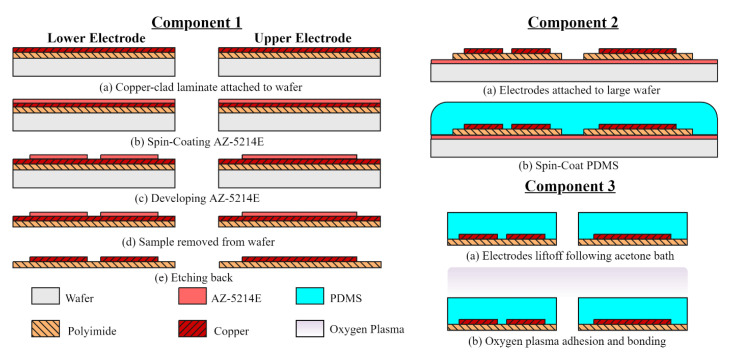
Capacitive sensor fabrication process. Component 1: defining the upper and lower electrode patterns. Component 2: developing the dielectric layer. Component 3: sensor assembly.

Soft lithography processes were utilized to fabricate the capacitive sensor. The sensor’s design contains three layers: an upper layer containing one electrode, followed by a dielectric layer, then four electrodes on the bottom layer. A brief overview of the device fabrication is illustrated in Figure 2 and is divided into three components.

The first component, denoted Component 1, deals with the construction of the upper and lower electrodes. Realized by employing an etch-back photolithography process. The electrodes are made from DuPont Pyralux AC092500EN (DuPont, Durham, NC, USA), which is an all-polyimide single-sided copper-clad laminate (polyimide thickness 25 µm, copper thickness 9 µm). The copper-clad film was diced to proper dimensions and attached to a glass handling wafer using Kapton tape. A standard degrease procedure using acetone, methanol, and deionized (DI) water (AMD) was used to remove oils and other contaminates. AZ-5214E positive photoresist was spun onto the substrate at a speed of 1200 rpm for 30 s to achieve a film thickness of 2 µm. The photoresist-coated film was soft baked at 100 °C for 60 s to reduce the residual solvent concentrations, then immediately patterned by means of contact printing using a UV g-line light source at $9\frac{mW}{cm^{2}}$. Afterward, the patterns were developed in a diluted AZ 400K solution (1 AZ 400K: 4 DI H_2_O) and agitated for 45 s to remove the softened photoresist. Next, the copper was etched in ferric chloride solution, defining the electrodes after approximately 15 min of agitation at room temperature using a magnetic stirrer.

The second component of the sensor fabrication process is developing the PDMS dielectric layer, detailed in Figure 2 as Component 2. This work chose Slygard^®^ 184 Silicone Elastomer Kit (Dow Corning, Midland, MI, USA) as the dielectric material. The PDMS was prepared with a 10:1 ratio of PDMS pre-polymer and curing agent. After thoroughly mixing with a spatula for 5 min. the mixture is degassed in a vacuum chamber to remove any air bubbles produced. A sacrificial layer of AZ-5214E photoresist was spun onto a 4 × 4-inch wafer for the electrodes to adhere to. This wafer size was chosen to prevent the edge bead from affecting device uniformity. Then, PDMS was spin coated at 700 rpm for 30 s. According to Lei et al., the thickness of the dielectric layer is a function of the spinning speed, which corresponds to approximately 120 µm [25]. After spin coating, the sample was cured on a hotplate for 4 h at 90 °C.

The third component of the fabrication process is the sensor assembly, denoted Component 3. Assembly was accomplished by PDMS-to-PDMS oxygen plasma bonding. Doing so prevents the need for a PDMS adhesion layer, significantly reducing dielectric uniformity issues. First, a cutout of the sample’s perimeter was made and the excess PDMS was removed. Followed by an acetone bath which dissolves the photoresist adhesion layer, lifting off the samples. The PDMS-coated electrodes were degreased and cleaned with isopropyl alcohol, then exposed to oxygen plasma at 80 W for 25 s, bonded, and heat-treated on a hotplate at 100 °C for 60 s. Introducing heat was necessary to increase the mobility of the active sites thereby increasing the probability of interactions necessary for successful PDMS-to-PDMS bonding.

Three tests were conducted to characterize the tactile sensor. First, the change in capacitance as a function of time was measured with an LCR meter (Keysight E4980A, Santa Rosa, CA, USA) to determine the sensor’s response speed. Second, the sensor was compressed along the negative z-direction using a strain gauge (MTS Landmark Servohydraulic Test System, Eden Prairie, MN, USA) with specially designed grips to determine the linear range and define the sensor’s sensitivity. Pressure measurements were conducted in the range of 0 kPa to 1765 kPa to evaluate the performance under loads expected in a RTSA prothesis. This load range was chosen thanks to the data provided by Farmer et al. where one can expect glenohumeral contact forces to reach up to 450 N [20]. Third, the sensor was mounted to a custom testing stage and interfaced with a signal conditioning circuit to monitor the change in signal over time.

**Figure 3:**
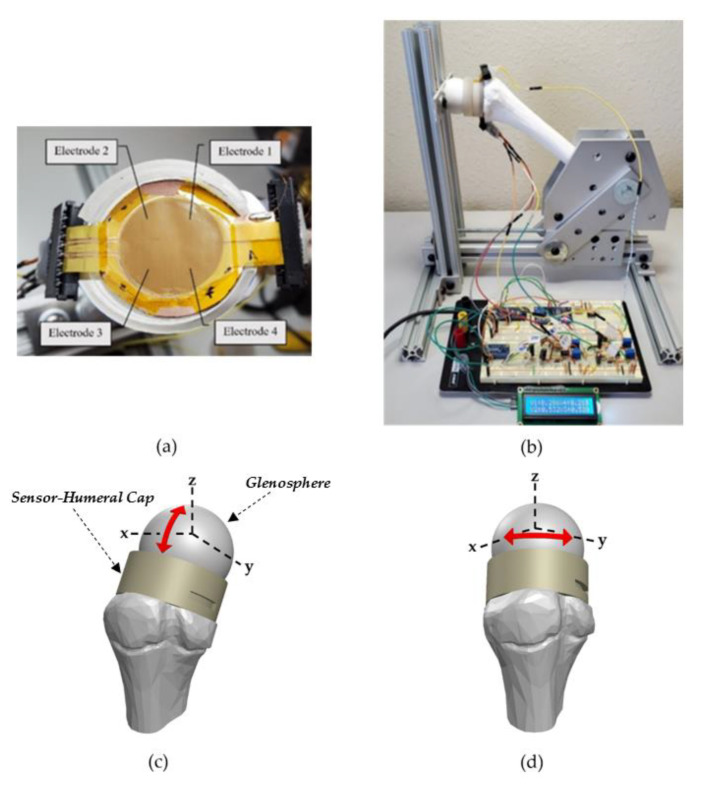
(**a**) Capacitive sensing array fixed inside the 3D printed humeral cap. (**b**) Testing configuration. (**c**) Sensor–humeral schematic highlighting the two positions used for results Section 3.3 (**d**) Sensor–humeral schematic highlighting the two positions used for results Section 3.3.

Detailed in Figure 3b is the arrangement to monitor pressure variations across the humeral–glenoid interface. The arrows shown in Figure 3c,d indicate the four sensor-humeral positions tested with respect to the glenosphere. The testing stage consists of a 3D printed humerus bone, glenosphere, and humeral cap which are manually pressed against a fixed glenosphere to obtain pressure measurements. Kapton tape anchors the tactile capacitive sensing array to a shield which is secured to the humeral cap via double-sided tape as shown in Figure 3a. A shield mitigates parasitic capacitance and any external interference. This configuration is connected in series with the data acquisition circuitry using FFC connectors. An Arduino nano is responsible for powering the system and acquiring the conditioned signal. A 15 kHz sinusoidal signal is produced by an AD9850 direct digital synthesis converter which is amplified by a non-inverting operational amplifier. Then, connected to the signal electrode to drive the sensor. A frequency of 15 kHz was chosen to reduce the inductance of the conditioning circuit, minimizing signal interference since there are overlapping leads. The capacitive sensor is connected in series with a potentiometer to form a capacitive-resistive network. Equation (4) provides the transfer function of this circuit:

(4) $$\left|\frac{V_{out}}{V_{ac}}\right|=\frac{\varpi R_{pot}C_{x}}{\sqrt{1+{\left(\varpi R_{pot}C_{x}\right)}^{2}}}$$

From Equation (1) we know that the capacitance increases as spacing between overlapping parallel plates decreases. Using this relationship and solving Equation (4), one can deduce that the output voltage is proportional to capacitance variations and therefore variations in pressure. The sensing electrodes are connected to the signal conditioning circuity which amplifies and rectifies the time-varying signal to produce a direct current waveform.

## 3. Results

### 3.1. Response Speed

**Figure 4:**
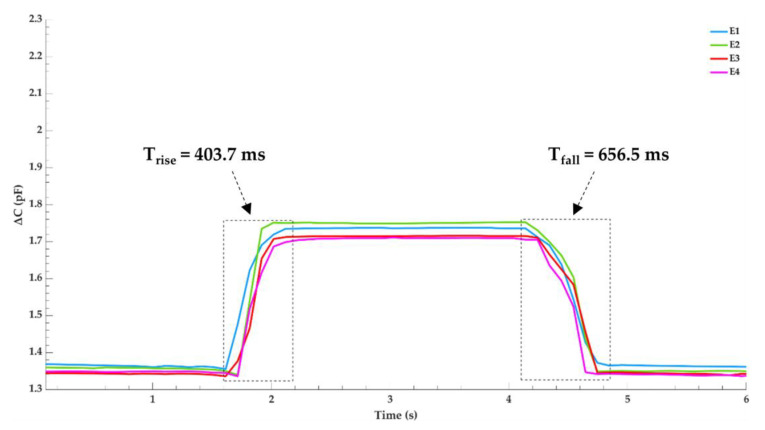
Pressure response and relaxation time of the sensor when a 14.13 kPa precision weight is quickly loaded and unloaded.

To evaluate the sensors response speed, a thin glass layer is placed on top of the sensor and a pressure of 14.13 kPa is quickly applied via a 100 g precision weight for approximately four seconds and then removed. A thin glass layer provides separation between the metal precision weight and ensures the load is evenly distributed across the electrodes surface area. An LCR meter is used to record the capacitance change for each electrode and the results are shown in Figure 4 which was plotted using MATLAB. To plot the true capacitance, the difference between capacitance with and without the glass layer is measured and subtracted from the final results. With no load applied, there is a maximum capacitance discrepancy of 23.66 fF between electrodes E1 and E4, indicating great sensor uniformity. Highlighted in the figure is the average response time for the loading and unloading process. When the sensor is loaded, it takes 403.7 ms to reach an average capacitance change of 0.384 pF. During this loading period, a maximum capacitance deviation of 39.71 fF occurs between electrodes E2 and E4. When pressure is unloaded, the sensor takes 656.5 ms to reach a stable value. A slower fall time is attributed to the pressure due to the gravity the glass layer places on the sensor as the PDMS relaxes back to its original state. Once the signals have stabilized, the relative capacitance for each electrode before and after loading remains unchanged. 

### 3.2. Sensitivity

**Figure 5:**
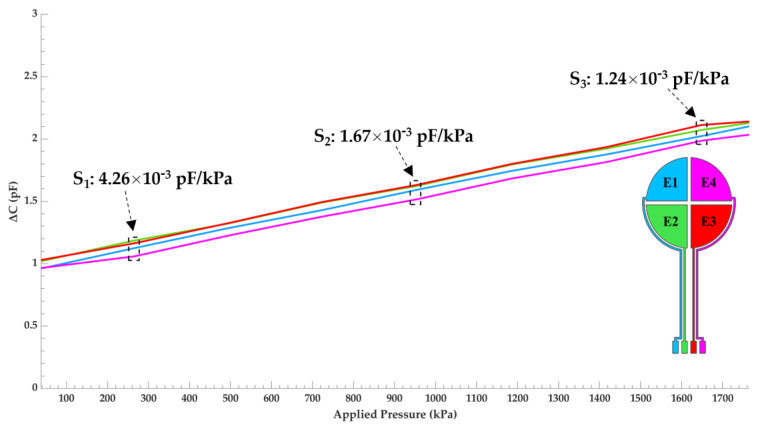
Capacitance change of the sensor as a function of pressure for each electrode. The plot is color coated to reflect the electrode’s location on the capacitive sensor’s schematic.

The sensitivity was determined by measuring the capacitance as a function of pressure for each of the four sensing electrodes with a strain gauge. Figure 5 details the outcome of said testing and was plotted using MATLAB. The plot is color coated to reflect each electrode’s location on the capacitive sensor’s schematic, located on the bottom right of the plot. The linear range of the sensor is observed between 37 kPa to 1765 kPa. A maximum capacitance change of 1.11 pF was measured at a pressure up to 1765 kPa. Variations of capacitance between each electrode remains small with 0.11 pF between electrodes E3 and E4 being the greatest. This deviation is a result of minute uniformity differences in the dielectric layer between electrodes. The sensitivity (pF/kPa) was calculated in three regions, computed using Equation (3), and is labeled in the figure. Overall, the sensitivity is rather uniform across the linear range with a max change of 3.02 × 10^−3^ pF/kPa between regions S_1_ and S_3_. Compared with similar capacitive sensors tested in this pressure region [25,26], the sensitivity of our device is very good with the highest measured to be 4.26 × 10^−3^ pF/kPa at a pressure of 250 kPa.

### 3.3. Signal Distribution

**Figure 6:**
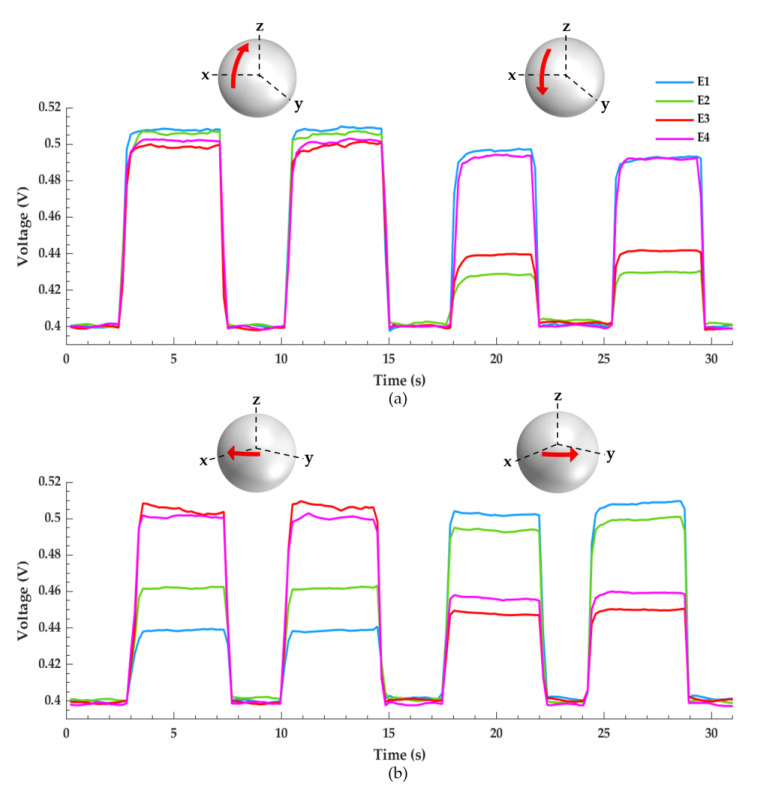
Pressure distribution across the four sensing electrodes as the sensor–humeris model is compressed against the glenosphere. (**a**) The first pair of waveforms show an almost uniform pressure distribution across the electrodes, indicating a fully abducted shoulder. The second pair of waveforms show that electrodes E1 and E4 experience the greatest magnitude of pressure, indicating a fully adducted shoulder. (**b**) The first pair of waveforms show that electrodes E3 and E4 experience the greatest magnitude of pressure, indicating a shoulder that is internally rotated. The second pair of waveforms show that electrodes E1 and E2 experience the greatest magnitude of pressure, indicating a shoulder that is externally rotated.

Figure 6a,b depicts the pressure distribution across the four sensing electrodes as the sensor–humeral model is compressed against the glenosphere in a right shoulder configuration using the testing setup shown in Figure 3b. These data portray the sensor’s potential to graphically analyze glenohumeral contact forces. The sensor–humeris model is statically held against the glenosphere in four different positions for approximately four seconds then returned to its initial position. Both figures measure two glenohumeral positions and each position is tested twice. A schematic is included for each waveform and the red arrows illustrate the sensor–humeris models’ approximate location with respect to the glenosphere. 

In Figure 6a, the signals are collected by statically pressing the sensor–humeris model along the glenosphere’ s positive and negative z-axis as detailed in Figure 3c. The first pair of waveforms are acquired by compressing the sensor–humerus model towards the positive z-direction, almost normal to the glenosphere to simulate a fully abducted shoulder. All four electrodes experience almost uniform pressure distribution with slightly more pressure focused anteriorly, i.e., electrodes E1 and E2. To obtain the second set of waveforms, the sensor–humeris model is held towards the negative z-direction, representing a sensor–humeris model that is medially held in a resting position to replicate a fully adducted shoulder. Here, sensing electrodes E1 and E4 experience the greatest magnitude of pressure showing a superior and proximally focused pressure distribution. In Figure 6b, the sensor–humeris model is translated along the glenosphere’ s positive x-axis and y-axis as detailed in Figure 3d. The first set of waveforms are captured as the sensor–humeris model is held towards the positive x-direction, medially and interiorly positioned, with a slight pitch upwards to simulate a shoulder that is internally rotated, showing an anterior pressure distribution as electrodes E3 and E4 experience the greatest magnitude of pressure. The second set of waveforms are collected as the sensor–humeris model is held towards the positive y-direction, fixed laterally towards the posterior edge of the glenosphere to simulate an externally rotated shoulder, where the greatest magnitude of pressure is focused on the posterior electrodes, E1 and E2.

Small signal discrepancies can be seen in all figures which are attributed to a few factors. The foremost discrepancy is the steady-state signal disparity between electrodes as no pressure is applied, which is a result of the initial pressure due to gravity the humeral cap applies across the sensor as it rests between the 3D printed humeral–glenoid interface. In addition, the uneven pressure variations caused by the user’s hand as the humeral cap is held against the glenosphere create jagged waveforms of up to 10 mV. Moreover, noise in the form of parasitic capacitance can suddenly drop the signal as shown in the first waveform of Figure 6b, electrode E3. 

## 4. Discussion

This work developed a tactile array capacitive sensor for potential use in RTSA utilizing four electrodes to characterize four components of the glenohumeral contact forces, which can easily be expanded to eight or more electrodes if necessary. This study provides proof of concept in our idea to employ a capacitive sensor to monitor variations in pressure, justifying additional research to improve the performance, capability, and reliability of the sensor. 

Results from Figure 4 confirm that the PDMS dielectric retracts back to its initial state following compressive deformation and the strategy employed to fabricate a uniform dielectric layer was successful. Great sensor linearity and sensitivity is observed in the target pressure range for a RTSA prothesis as shown in Figure 5. The data acquisition circuitry built in-house successfully captured variations in signal from each electrode which correlates to the pressure distribution across the humeral–glenoid interface. However, using a hand to manually position the sensor–humerus model to apply pressure against the glenosphere can create inference with the input and output leads creating parasitic capacitive noise. Additionally, a non-structured PDMS dielectric film greatly reduces the sensors response speed [23]. 

Future work will focus on biocompatible friendly materials, sensor optimization, and calibration to match loads seen in RTSA. While PDMS is biocompatible, copper is not. Thus, a biocompatible friendly material such as titanium should be used instead of copper for electrode fabrication. The sensitivity can be further improved by increasing the dielectric permittivity by infusing ceramics such as barium titanate nanoparticles into the dielectric layer, doubling the sensitivity of the sensor [27]. The response speed of the sensor can be greatly improved by modifying the dielectric layer and surface area of the electrodes. Utilizing carbon nanotube electrodes fabricated with microstructures such as a micro pyramid array increases the sensors sensitivity, response speed, and offers high recoverability and stability [28,29]. Furthermore, altering the overlapping area between the top and bottom electrodes can enable normal and shear stress measurements [30]. To overcome the signal interference during testing, flexible ribbon cables with adequate shielding will replace the wiring to interface the sensor with the data acquisition circuitry. Lastly, studies must be conducted to evaluate the mechanical fatigue of the sensor to ensure it can be integrated into the human body for long term joint load monitoring, as 91% of patients under 60 years old with an RTSA prothesis have a 10-year implant survival rate [31]. 

## 5. Conclusions

This work introduces a flexible capacitive tactile sensor with the potential to monitor several forces in an RTSA prosthesis. A PDMS dielectric was chosen due to its biocompatibility and tunable elasticity, prepared with a 10:1 mixing ratio, along with four sensing electrodes and a single source electrode fabricated from a copper-clad laminate. Great uniformity is observed between electrodes with a maximum capacitance deviation of 23.66 fF when no load is applied. When a pressure of 14.13 kPa is applied via a 100 g precision weight, the sensors maximum response speed is 403.7 ms. A maximum capacitance change of 1.11 pF is recorded between the target pressure range of 37 kPa to 1765 kPa. In this range, great linearity is observed and a sensitivity of 1.24 × 10^−3^ pF/kPa is recorded at a load of 1649 kPa. A testing stage is built and components of the RTSA prothesis are 3D printed to determine if a capacitive sensor can be feasibly implemented to measure glenohumeral contact forces. A data acquisition circuit is designed to monitor these forces between the 3D printed glenoid and humeral cap. Then, a graphical description of the results is provided to establish a proof of concept that pressure variations can be presented in a comprehensible manner, providing the foundation that future prototypes will build upon fabrication of a sensor which is optimized for RTSA procedures.

## Data Availability

Not applicable.
